# OD01 Delays In Funded Access To Medicines: A Global Perspective

**DOI:** 10.1017/S0266462324001399

**Published:** 2025-01-07

**Authors:** Mah Laka, Yuan Gao, Drew Carter, Tracy Merlin

## Abstract

**Introduction:**

There are significant delays in the funded access to medicines. Studies indicate that in many countries it takes more than a year for patients to have funded access to medicines after market authorization. This study aimed to understand the disparities in timelines for funded access to medicines across different countries and to identify underlying reasons for this access gap.

**Methods:**

We conducted a scoping review to examine the nature of health technology assessment (HTA) processes, current methods, and policies for medicines in ten jurisdictions. The jurisdictions included in this study are Australia, Canada, France, Germany, South Korea, the Netherlands, United Kingdom (divided into England, Scotland and Wales), and United States of America. The information was extracted from the websites of International Network of Agencies for Health Technology Assessment (INAHTA) member agencies in the selected jurisdictions, grey literature from governments’ websites, and peer-reviewed literature.

**Results:**

Overall median time from submission of the evidence dossier to HTA recommendations for most jurisdictions is 22 weeks. Although there are similarities in the time taken to reach a funding decision, there are considerable variations in the time taken for patients to have funded access to medicines after HTA recommendations. Only a few countries mentioned a specific timeline within which medicines approved for funding should be listed. Time taken for price negotiations and other arrangements (i.e., risk-sharing agreements) may contribute to varying timelines for listing medicines for funding. Mostly, such negotiations are confidential and may not be time limited.

**Conclusions:**

There was surprising consistency, globally, in the time it takes for funding decisions after medicines registration. The causes of delays in the medicines’ listing decisions are multifactorial and mostly occur after HTA recommendations. The parallel regulatory-assessment process and prioritization tend to reduce the time to a funding decision. However, transparency is needed in the listing process to improve overall timeliness.

